# Efficacy of a HER2-Targeted Thorium-227 Conjugate in a HER2-Positive Breast Cancer Bone Metastasis Model

**DOI:** 10.3390/cancers15133419

**Published:** 2023-06-29

**Authors:** Jenny Karlsson, Urs B. Hagemann, Véronique Cruciani, Christoph A. Schatz, Derek Grant, Christine Ellingsen, Alexander Kristian, Shirin Katoozi, Dessislava Mihaylova, Steinar R. Uran, Mari Suominen, Roger M. Bjerke, Olav B. Ryan, Alan Cuthbertson

**Affiliations:** 1 Targeted Radiopharmaceuticals, Bayer AS, 0283 Oslo, Norway; veronique.cruciani@bayer.com (V.C.); grantderek38@gmail.com (D.G.); christineellingsen@gmail.com (C.E.); alexandr.kristian@gmail.com (A.K.); shirin.katoozi@bayer.com (S.K.); dessislava.mi@gmail.com (D.M.); steinar.uran@bayer.com (S.R.U.); roger.bjerke@bayer.com (R.M.B.); olav.ryan@bayer.com (O.B.R.); alan.cuthbertson@bayer.com (A.C.); 2 Bayer AG, 13342 Berlin, Germany; urs.hagemann@bayer.com (U.B.H.); christoph.schatz@bayer.com (C.A.S.); 3 Pharmatest, 20520 Turku, Finland; mari.suominen@pharmatest.com

**Keywords:** targeted alpha therapy, HER2-positive breast cancer, HER2-targeted therapy, thorium-227, bone metastasis, targeted radiotherapy, targeted thorium conjugate, intratibial mouse model

## Abstract

**Simple Summary:**

Human epidermal growth factor receptor 2 (HER2) is a protein present in increased amounts in breast cancer compared with healthy breast tissue. Anti-cancer drugs directed at HER2 can therefore kill cancer cells while reducing harmful effects to adjacent normal cells that do not have HER2. HER2-positive breast cancers often spread to bone as the disease progresses, resulting in incurable bone metastases, bone fractures and breaks, and severe pain for the patient. We have developed HER2-TTC (HER2-targeted thorium-227 conjugate), an intravenously injected cancer therapy that delivers lethal radiation to HER2-positive cancer cells. We tested HER2-TTC in cell culture and mouse models, including those that mimic HER2-positive human breast cancer metastases in bone. In the in vivo models, HER2-TTC treatment killed breast cancer cells and prevented cancer-induced abnormal changes in bone. These results suggest that HER2-TTC treatment could be beneficial to patients with HER2-positive breast cancer with bone metastases.

**Abstract:**

Human epidermal growth factor receptor 2 (HER2) is overexpressed in 15–30% of breast cancers but has low expression in normal tissue, making it attractive for targeted alpha therapy (TAT). HER2-positive breast cancer typically metastasizes to bone, resulting in incurable disease and significant morbidity and mortality. Therefore, new strategies for HER2-targeting therapy are needed. Here, we present the preclinical in vitro and in vivo characterization of the HER2-targeted thorium-227 conjugate (HER2-TTC) TAT in various HER2-positive cancer models. In vitro, HER2-TTC showed potent cytotoxicity in various HER2-expressing cancer cell lines and increased DNA double strand break formation and the induction of cell cycle arrest in BT-474 cells. In vivo, HER2-TTC demonstrated dose-dependent antitumor efficacy in subcutaneous xenograft models. Notably, HER2-TTC also inhibited intratibial tumor growth and tumor-induced abnormal bone formation in an intratibial BT-474 mouse model that mimics breast cancer metastasized to bone. Furthermore, a match in HER2 expression levels between primary breast tumor and matched bone metastases samples from breast cancer patients was observed. These results demonstrate proof-of-concept for TAT in the treatment of patients with HER2-positive breast cancer, including cases where the tumor has metastasized to bone.

## 1. Introduction

Targeted alpha therapy (TAT) takes advantage of the combination of the highly potent radiobiological properties of an alpha-particle-emitting radionuclide combined with a tumor-targeting moiety, such as a monoclonal antibody [1,2,3,4]. The primary advantage of alpha particle emitters over other types of radiation is their high linear energy transfer (LET) [5], high relative biological effectiveness (RBE) [6,7], and limited collateral damage to adjacent normal tissues due to the short (<100 µm) range of the alpha particles. The mechanism of cytotoxicity of TAT is based on the generation of a dense ionizing track of the alpha particle, inducing difficult-to-repair, clustered DNA double strand breaks (DSBs) [8,9,10]. As we have previously demonstrated, alpha particle emitters induce phosphorylation of the histone protein H2AX (γ-H2AX) and G2/M cell cycle arrest in various cancer cell lines, indicating the involvement of the DNA damage response [2,11,12].

**Figure 1:**
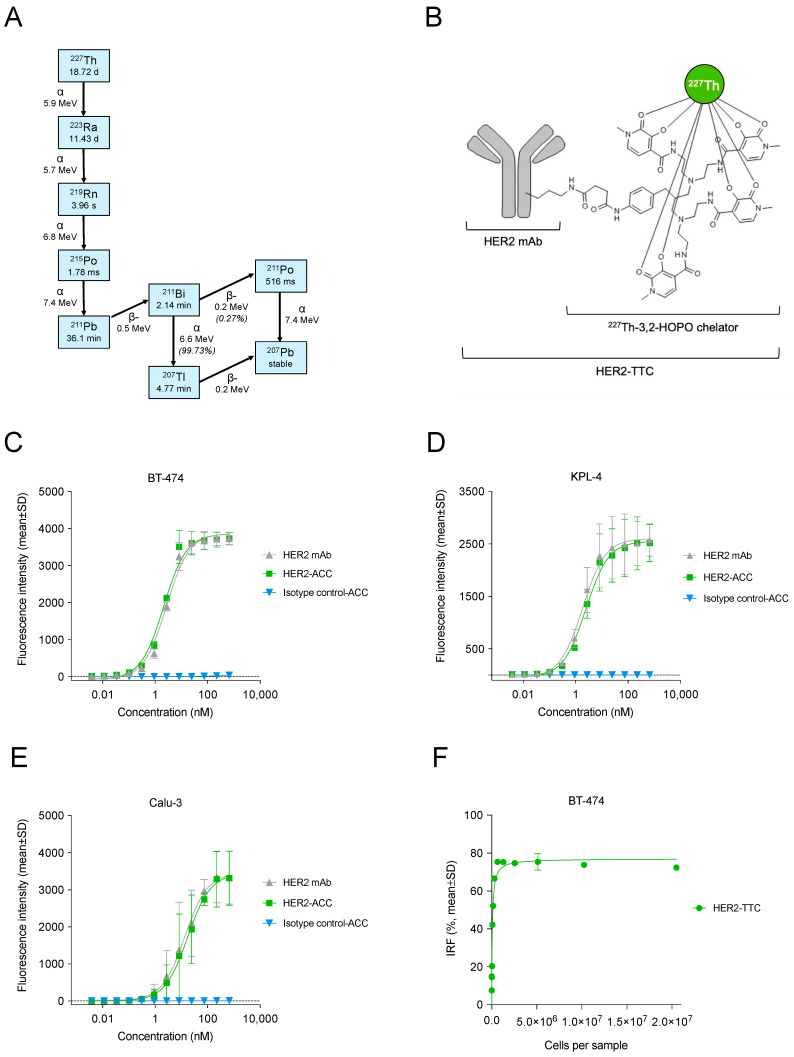
Structure, binding, and stability of HER2-TTC. (**A**) Decay chain of thorium-227. Thorium-227 decays via its alpha- and beta-emitting daughters to stable non-radioactive lead-207 (modified from [18]). (**B**) Structure of HER2-TTC consisting of the HER2 mAb covalently attached to the 3,2-HOPO chelator enabling efficient radiolabeling with thorium-227 (modified from [29]). (**C**–**E**) Binding of HER2 mAb, HER2-ACC, and non-radiolabeled isotype control in HER2-expressing (**C**) BT-474 and (**D**) KPL-4 human breast cancer cells and (**E**) Calu-3 human lung cancer cells, as determined via flow cytometry (*n* = 2–4). (**F**) Immunoreactive fraction (IRF) curve showing efficient binding of HER2-TTC to HER2 receptors in BT-474 cells (*n* = 1–3).

Radium-223 dichloride (radium-223, Xofigo^®^) is the first-in-class alpha-emitting radiopharmaceutical approved by the US Food and Drug administration (FDA) and the European Medicines Agency (EMA) for the treatment of patients with castration-resistant prostate cancer (CRPC) with symptomatic bone metastases but no known visceral metastases [13,14,15]. Radium-223 is a calcium-mimetic that selectively binds to the hydroxyapatite in bone and preferentially targets areas of active bone metabolism, such as bone metastases, where the emitted alpha radiation disrupts the activity of both bone and cancer cells [15,16,17]. In preclinical in vitro and in vivo experiments, radium-223 has been shown to directly inhibit the proliferation of cancer cells in both osteolytic breast cancer and osteoblastic prostate cancer bone metastases and to inhibit the differentiation of osteoblasts and osteoclasts due to both its physical bone-homing properties and its active incorporation by osteoblasts [15]. The lack of suitable chelating agents for radium-223 has limited its use for TATs, whereas thorium-227 (half-life of 18.7 days), the parent radionuclide of radium-223 (Figure 1A), offers promise as a wider-ranging alternative due to the availability of efficient chelators, such as 3,2-hydroxypyridinone (3,2-HOPO) [10,18,19]. The 3,2-HOPO chelator can be conjugated to a range of targeting moieties, such as antibodies, delivering the radiation dose specifically to cancer cells, and thereby minimizing exposure of the normal tissue [20,21]. We have previously described the properties of several targeted thorium-227 conjugates (TTCs) binding to a variety of tumor antigens in various cancers [2,10,11,19,22,23,24,25,26,27,28,29].

Human epidermal growth factor receptor 2 (HER2) is overexpressed in 15–30% of breast cancers, and it is associated with poor prognosis and treatment outcome [30,31]. Currently, the first-line treatment of advanced HER2-positive breast cancer includes dual blockade of HER2 with the tyrosine kinase inhibitors trastuzumab and pertuzumab in combination with systemic chemotherapy with a taxane or vinorelbine [31,32,33,34,35,36]. HER2-positive breast cancer typically metastasizes to bone, affecting nearly all patients with end-stage disease [37,38]. Despite several treatment options available for patients with bone metastases, breast cancer bone metastases are incurable and associated with significant morbidity and mortality [37,38,39]. Therefore, new strategies for HER2-targeting therapy are needed to improve the quality of life and life expectancy of patients with HER2-positive breast cancer metastasized to bone.

Here, we present the preclinical characterization of a HER2-targeting thorium-227 conjugate (HER2-TTC), which is based on the amino acid sequence of the humanized IgG1 antibody trastuzumab covalently conjugated to a thorium-227-complexing 3,2-HOPO chelator. HER2-TTC demonstrated potent in vitro cytotoxicity in various HER2-positive cancer cell lines and strong in vivo antitumor efficacy both in subcutaneous KPL-4 and Calu-3 xenograft models and in an intratibial BT-474 mouse model mimicking breast-cancer-induced metastatic bone disease. Additionally, we compare for the first time the HER2 expression of matched patient samples of primary breast tumors and bone metastases. Taken together, our results demonstrate proof-of-concept for TAT in the treatment of patients with HER2-positive breast cancer metastasized to bone.

## 2. Materials and Methods

### 2.1. Compounds

A HER2-targeting antibody based on the trastuzumab sequence (HER2 mAb), HER2-TTC, the radiolabeled isotype control, the non-radiolabeled HER2-targeted antibody-chelator conjugate (HER2-ACC), and the radionuclide thorium-227 were manufactured by Bayer AG (Wuppertal, Germany) or Bayer AS (Oslo, Norway). The vehicle used in all in vivo studies was 30 mM citrate, 70 mM NaCl, 0.5 mg/mL para-aminobenzoic acid (PABA), 2 mM ethylenediaminetetraacetic acid (EDTA; pH 5.5), supplemented with 0.1 mg/mL IgG2a-κ antibody from murine myeloma (monoclonal UPC10 antibody, Sigma-Aldrich, Burlington, MA, USA).

### 2.2. Cell Culture

BT-474 and SK-BR-3 human breast cancer cells and Calu-3 human lung cancer cells were obtained from ATCC (American Type Culture Collection, Manassas, VA, USA) and KPL-4 human breast cancer cells from the Kawasaki Medical School (Kurashiki, Japan) and cultivated according to supplier protocols at 37 °C in a humidified atmosphere containing 5% CO_2_. The cells were regularly subjected to DNA fingerprinting at DSMZ (Deutsche Sammlung von Mikroorganismen und Zellkulturen, Braunschweig, Germany) and tested to be free from mycoplasma contamination using MycoAlert (Lonza) directly before use.

### 2.3. Synthesis and Characterization of HER2-TTC

The non-radiolabeled HER2-ACC was prepared through conjugating an N-hydroxysuccinimide-activated 3,2-HOPO chelator covalently to the ε-amino groups of the lysine residues of the HER2 mAb, as previously described [20,21]. A chelator-to-antibody ratio of 0.8 was determined via HPLC. Thorium-227 was purified from an actinium-227 generator as previously described [40] and HER2-ACC was radiolabeled with thorium-227 in citrate buffer (pH 5.5) at +22 °C for 60 min as previously described [29], resulting in HER2-TTC (Figure 1B). A non-binding radiolabeled isotype control was prepared similarly to HER2-TTC. Radiochemical purity (RCP), defined as the amount of thorium-227 bound to the HER2-TTC, was determined via instant thin-layer chromatography (iTLC). HER2-TTC was analyzed for radiostability over the course of 48 h via HPLC.

Binding properties of the HER2 mAb, HER2-ACC, and isotype control-ACC were assessed in HER2-expressing BT-474, KPL-4, and Calu-3 cells. Determination of EC_50_ values and antibodies bound per cell (ABC) were performed via flow cytometry as previously described [29]. Briefly, 80% confluent cells were harvested in Accutase solution (Biowest, Nuaillé, France), filtered through 40 µm cell strainers and washed with phosphate-buffered saline (PBS). The cells were counted, suspended in flow buffer (PBS supplemented with 1–5% fetal bovine serum [FBS]), and transferred to a V-shaped 96-well plate at a density of 50,000–100,000 cells/well. All following steps were performed at 4 °C. Serial dilutions (1:3) of the HER2 mAb, HER2-ACC, or isotype control-ACC were added to the cells at a starting concentration of 100 µg/mL (667 nM) and incubated for 1 h. Cells were washed twice with flow buffer and bound test compounds were detected through incubating the cells with a 1:50 dilution of R-phycoerythrin (PE)-labeled anti-human IgG Fc antibody (BioLegend, San Diego, CA, USA) for 45–60 min. After a final washing step, the median fluorescence intensity (MFI) was determined using a Guava^®^ easyCyteTM flow cytometer (Luminex, Austin, TX, USA) and analyzed using the FlowJo software. EC_50_ values were calculated based on the measured MFIs using the GraphPad Prism software. ABCs were determined using the QuantibriteTM PE kit (BD Biosciences, San Jose, CA, USA) according to the manufacturer’s protocol.

The immunoreactive fraction (IRF) of HER2-TTC, indicative of the binding affinity of the radiolabeled product, was determined as described by Lindmo [41]. Briefly, BT-474 cells were harvested using Accutase solution and suspended in DMEM/Ham’s F12 cell culture medium (Biowest). The cells were washed once and re-suspended in IRF buffer (PBS supplemented with 3% bovine serum albumin [BSA]). A dilution series with 20.5 × 10^6^–5 × 10^3^ cells in a final volume of 195 µL was prepared. A total of 10 µL of IRF buffer was added to all samples. To assess nonspecific binding, separate samples were treated with a 10 µL excess of the HER2 mAb prior to the addition of HER2-TTC. All samples were incubated on a shaker at 750 rpm and 37 °C for 1 h. HER2-TTC was diluted in IRF buffer at a concentration of 10 Bq/µL, 10 µL (=100 Bq) was added to all samples, and the samples were incubated on a shaker at 750 rpm and 37 °C for 1 h. Next, the samples were centrifuged and 100 µL aliquots of the supernatants were transferred into new microcentrifuge tubes. Subsequently, the radioactivity in all supernatants and cell samples was measured on an automated germanium detector. The IRF of HER2-TTC, expressed as the percentage of the total radiolabeled population, was estimated through plotting standard saturation binding.

### 2.4. In Vitro Cytotoxicity of HER2-TTC

In vitro cytotoxicity experiments were performed after a 5-day exposure to 10, 20 or 40 kBq/μg HER2-TTC or radiolabeled isotype control in BT-474, KPL-4, and Calu-3 cells using the CellTiter Glo^®^ cell (CTG) viability assay (Promega, Madison, WI, USA). One day before the experiments, subconfluent cells were harvested, counted, and seeded into 384-well plates at a density of 500–2000 cells/well in 30 µL of cell culture medium. The cells were incubated at 37 °C in a humidified atmosphere with 5% CO_2_ overnight. A 0.0001–20 kBq/mL dilution series of HER2-TTC or radiolabeled isotype control (10, 20 or 40 kBq/μg) was added to the samples, and the samples were incubated at 37 °C/5% CO_2_ for five days. Cell viability was determined using the CTG assay and luminescence was measured on an EnVision plate reader (PerkinElmer, Waltham, MA, USA). The viability of treated cells was expressed as % of control cells (cultured in cell culture medium only) and half-maximal inhibitory concentrations (IC_50_ in kBq/mL) were determined via four-parameter logistic curve-fitting using the GraphPad Prism software.

### 2.5. In Vitro Mode-of-Action of HER2-TTC

To investigate the mode of action of HER2-TTC, DNA double-strand break (DSB) formation and cell cycle distribution were determined in HER2-TTC-treated BT-474 cells.

DSB formation was determined through measuring the percentage of phosphorylated histone protein H2AX (γ-H2AX)-positive BT-474 cells upon HER2-TTC treatment. One day before the experiments, BT-474 cells were seeded in 96-well plates (10,000 cells/well in 100 µL of cell culture medium) and incubated at 37 °C in a humidified atmosphere with 5% CO_2_ overnight. HER2-TTC or radiolabeled isotype control at 0.07–50 kBq/mL was added to the wells and the samples were incubated for 24, 48, or 72 h. Then, the cells were washed with PBS, fixed with 4% paraformaldehyde (PFA) at room temperature for 10–30 min, and permeabilized with 0.2% Triton^TM^ X-100 in PBS at room temperature for 15–30 min. After 2–3 washes with PBS, the cells were incubated with the Alexa Fluor^®^ 647 anti-H2A.X phospho (Ser139) antibody (1:200; BioLegend) and Hoechst 33342 nucleic acid stain (1 µg/mL in PBS supplemented with 3% BSA; Invitrogen, Waltham, MA, USA) at 4 °C for 16–44 h. After the staining, images were acquired on the Operetta CLS analysis system (PerkinElmer, Harmony software, 5× objective and 40× water objective) and analyzed for the induction of DSBs through measuring the percentage of γ-H2AX-positive cells.

For the cell cycle analysis, BT-474 cells were seeded in 6-well plates (40,000 cells/well in 4 mL of cell culture medium) and incubated overnight at 37 °C/5% CO_2_. The cells were exposed to HER2-TTC or radiolabeled isotype control at 0.5 kBq/mL for 24, 48, or 72 h. After the treatment, the cells were washed, detached, and harvested in PBS at 4 °C. Next, the cells were fixed and permeabilized with 70% ethanol at −20° C for 30 min, followed by washes with plain PBS and PBS supplemented with 3% FBS. Subsequently, the cells were mixed with FxCycleTM PI/RNase staining solution (Invitrogen) and analyzed on a Guava^®^ easyCyteTM flow cytometer using a gating strategy where first only single cells were selected, followed by gating of sub-populations of cells in G1, S, or G2/M phases. For each treatment, the proportion of cells in G1, S or G2/M phases was determined.

### 2.6. In Vivo Efficacy of HER2-TTC in Various Xenograft Models

The in vivo anti-tumor efficacy of HER2-TTC was evaluated in the subcutaneous (s.c.) KPL-4 breast cancer and Calu-3 lung cancer xenograft models and in the intratibial BT-474 mouse model mimicking breast cancer metastasized to bone.

HER2-TTC was formulated in 30 mM citrate supplemented with 70 mM NaCl, 0.5 mg/mL PABA, and 2 mM EDTA. To prevent rapid clearance of the test compound, all mice were pre-dosed with 150–210 μg of an irrelevant mouse antibody (IgG2a-κ) one day before TTC treatment [42].

In the KPL-4 efficacy study, female athymic nude mice (Hsd: Athymic Nude-*Foxn1nu*, 4–5 weeks, Harlan) were inoculated s.c. with 1 × 10^6^ KPL-4 cells suspended in PBS. On day 14 after inoculation, the mice were randomized into control and treatment groups (*n* = 10) when KPL-4 tumors reached an average size of 11 mm^3^. KPL-4 tumor-bearing mice were treated with a single intravenous (i.v.) dose of vehicle (30 mM citrate, 70 mM NaCl, 0.5 mg/mL PABA, 2 mM EDTA, 0.075% PS-80, pH 5.5), radiolabeled isotype control (250 kBq/kg), or HER2-TTC at 100, 250, or 500 kBq/kg (total antibody dose 0.14 mg/kg). The study was terminated on day 31 after tumor inoculation.

In the Calu-3 efficacy study, female BomTac: NMRI-*Foxn1nu* mice (Taconic M&B A/S, Denmark) were inoculated s.c. with 5 × 10^6^ Calu-3 cells suspended in Matrigel^®^ Growth Factor Reduced (Sigma-Aldrich). On day 8 after inoculation, the mice were randomized into control and treatment groups (*n* = 10) when Calu-3 tumors reached an average size of 78 mm^3^. Calu-3 tumor-bearing mice were treated with a single i.v. dose of vehicle (30 mM citrate, 70 mM NaCl, 0.5 mg/mL PABA, 2 mM EDTA, 0.075% PS-80, pH 5.5), radiolabeled isotype control (250 kBq/kg), or HER2-TTC at 125, 250, or 500 kBq/kg (total antibody dose 0.14 mg/kg). The study was terminated on day 44 after tumor inoculation.

In the BT-474 efficacy study, female athymic nude mice (Hsd: Athymic Nude-*Foxn1nu*, 5–6 weeks, 17–30 g; Envigo, Huntingdon, UK) were inoculated with 1 × 10^6^ BT-474 cells (suspended in PBS) into the bone marrow of the right proximal tibia [43,44]. Six days after the cell inoculation, the mice were randomized into control and treatment groups (*n* = 9–12) according to body weight and treated with a single i.v. dose of HER2-TTC at 250 or 500 kBq/kg (total antibody dose 0.14 mg/kg). The control group received a single i.v. injection of 0.9% saline as vehicle control. The study was terminated on day 14, 28, or 42 after tumor inoculation.

Animal experiments were performed under the national animal welfare laws in Finland, Norway, and Germany and approved by the local authorities. Tumor growth and the development of tumor-induced changes in bone were monitored via X-ray imaging, micro-computed tomography (micro-CT), alpha camera imaging, gamma counting, and immunohistochemistry. In addition, systemic changes were monitored via analyzing the bone formation marker N-terminal procollagen type 1 (PINP) levels in serum. Animal body weight was monitored twice weekly as a measure of disease progression and possible treatment-related toxicity. At study termination, the animals were sacrificed via cervical dislocation under CO_2_ anesthesia.

### 2.7. In Vivo Mode-of-Action Analyses in the Calu-3 Model

To elucidate the in vivo mode of action of HER2-TTC, HER2 expression and H2AX phosphorylation was evaluated in tissue sections of formalin-fixed, paraffin-embedded (FFPE) Calu-3 tumors (*n* = 3) at the end of the study on day 37 after treatment with vehicle, 250 kBq/kg radiolabeled isotype control, or 250 kBq/kg HER2-TTC. HER2 expression was determined with the same method that was used for the detection of HER2 expression in patient samples (see below). H2AX phosphorylation was determined in FFPE tissue sections using the mouse anti-phospho-histone H2A.X (Ser139) antibody (1 μg/mL; clone JBW301, Merck Millipore, Darmstadt, Germany) at room temperature for 60 min, followed by incubation with the horseradish peroxidase (HRP)-labeled anti-mouse polymer (Dako). Immunoreactions were visualized using 3,3′-diaminobenzidine (DAB) as a substrate. Positive γ-H2AX signals were quantified using the HS Analysis Webkit tool (HS Analysis GmbH).

### 2.8. Radiography and Micro-CT Analyses in the BT-474 Model

The development of tumor-induced formation of abnormal bone was monitored via X-ray imaging before treatment start on day 5 after tumor inoculation and on days 14, 28, and 42. The mice were positioned on their ventral side, hind limbs stretched out, and X-ray images were taken with the Faxitron MX-20 D12 Cabinet X-ray System (Faxitron, Tucson, AZ, USA) using the Faxitron Dicom 3.0 software. At least one X-ray image (both hind limbs) per mouse was taken at each timepoint (34 kV, 7 s, magnification 2×). The lesion area and number of lesions in the hind limbs were determined from the images using the MetaMorph image analysis software (Molecular Devices, San Jose, CA, USA).

Micro-CT imaging of the proximal and mid-diaphyseal tibias was performed using the Skyscan 1072 micro-CT scanner (50 kV tube voltage, 201 µA X-ray intensity, 7 µm spatial resolution; Bruker, Billerica, MA, USA) with an average scan duration of 69 min. One tumor-bearing tibia from each treatment group (vehicle; 250 kBq/kg HER2-TTC; 500 kBq/kg HER2-TTC) was scanned and representative 3D images were reconstructed using the SkyScan NRecon software (version 1.6.10.4) and visualized using the SkyScan CTVox software (version 3.3.0).

### 2.9. Alpha Camera Imaging and Gamma Counting in the BT-474 Model

For the alpha camera imaging, mid-sagittal sections from non-tumor-bearing and BT-474 tumor-bearing tibias from mice treated with 250 kBq/kg or 500 kBq/kg HER2-TTC were obtained via methylmethacrylate (MMA) embedding and sectioning 14 days after tumor inoculation. For histologic comparison of each tibia, two sections were stained with hematoxylin and eosin (H&E) or Masson-Goldner Trichrome (MGT) stain, and one unstained section was used for alpha-camera imaging. The imaging system consisted of a ImagEM X2-1K EM-CCD camera (1024 × 1024 matrix pixel size, quantum efficiency >90% for 480–700 nm wave lengths, 12,000 electronic magnification; Hamamatsu Photonics, Hamamatsu City, Shizuoka, Japan) equipped with a f/0.95 focus lens assembled inside a dark box to ensure complete darkness. The CCD chip was cooled down to −50 °C to reduce image noise. Scintillation sheets with a thin uniform layer of silver-activated zinc-sulfide phosphor (ZnS:Ag, EJ-440, Scionix) were used to visualize individual scintillations caused by alpha particles. For alpha camera imaging, specimen glasses were placed with the section side towards the scintillation sheet, and for each set of samples, images were acquired for 50–55 h (frame duration 30 s, 5000–6600 frames in total). For image generation, single alpha radiation events were reconstructed from the individual frame images using the MATLAB software. Briefly, single frame images were averaged with a 51 × 51 kernel to obtain the background values which were then subtracted from the original image and then averaged again with a 5 × 5 kernel. Single radiation events that exceeded an image intensity threshold of 220 were recorded, regardless of the total signal intensity, and the position of the centroids was recorded. The resulting images include all such centroids from the individual images.

To assess HER2-TTC uptake, non-tumor-bearing and BT-474 tumor bearing tibias of mice treated with 250 kBq/kg or 500 kBq/kg HER2-TTC were collected 14, 28, or 42 days after tumor inoculation and the incorporated radioactivity was measured using an automatic gamma counter (Hidex, Turku, Finland).

### 2.10. Histology and Histomorphometry Analyses in the BT-474 Model

Histology and histomorphometry analyses were performed as previously described [3,45]. Briefly, tumor-bearing tibias were collected into 4% PFA for histological analysis of tumor and bone area and HER2 expression. Mid-sagittal sections were obtained from each tumor-bearing tibia, and if tumor tissue was not observed in the mid-sagittal section, a second set of sections was obtained. The sections were stained using H&E + Orange G and MGT staining and scanned with a digital slide scanner. Trabecular and cortical bone areas as well as tumor areas were measured from the H&E + Orange G -stained sections. The region of interest (ROI) for the bone area measurements was set in a standardized fashion and the bone area was determined according to color threshold using the CaseViewer software (3DHISTECH, Budapest, Hungary). Total and trabecular bone areas were determined through drawing separate ROIs, and cortical bone area was calculated through subtracting the trabecular area from the total bone area. Tumor area within the trabecular ROI and the whole section were drawn separately. HER2 expression was evaluated via immunohistochemical (IHC) staining with the anti-HER2 SP3 rabbit monoclonal antibody, (1:75 dilution; Spring Biosciences, Pleasanton, CA, USA). The HER2 status of the tumors was evaluated based on the membrane staining completeness and intensity according to the ASCO CAP 2018 guidelines [46] and analyzed using the ImmunoMembrane web application (https://biii.eu/immunomembrane, accessed on 4 August 2016). The HER2 status was scored on a scale from 0 to 3+ as follows: 0/1+ = negative, 2+ = equivocal, 3+ = positive.

In addition, one tibia section was subjected to von Kossa staining to demonstrate calcium deposits in the bone tissue. The tibia section was treated with 2.5% silver nitrate solution (AgNO_3_, Sigma Aldrich) for 10 min and exposed to UV light for 20 min. The section was rinsed several times with distilled water and incubated with 250 µL of 2.5% sodium thiosulfate (Merck Millipore) for 5 min to remove unreacted silver. Then, the tissue section was rinsed with distilled water and counterstained with 250 µL of nuclear fast red (Sigma-Aldrich) for 5 min and rinsed again with distilled water. Finally, the section was dehydrated through immersion in a graded series of alcohol solutions, cleared in xylene, and mounted using a mounting medium. Calcium deposits, visualized as light-reduced metallic silver, were detected using a light microscope with 10× magnification.

### 2.11. Biochemical Marker Analysis in the BT-474 Model

Serum samples were collected from the saphenous vein of the mice one day before tumor inoculation and on days 14, 28, and 42 after tumor inoculation, prepared within one hour of sampling, and stored at −80 °C for further analyses. Serum levels of the bone formation marker procollagen type I N-terminal propeptide (PINP) were measured using the Rat/Mouse PINP enzyme immunoassay (IDS Ltd., Tyne&Wear, UK). Absorbance at 450 nm was read with a VICTOR2^TM^ Multilabel counter (PerkinElmer).

### 2.12. Detection of HER2 Expression in Patient Samples

Anonymized and matched tumor and bone metastasis sample pairs from consenting breast cancer patients were analyzed for HER2 expression via IHC staining. HER2 expression was determined immunohistochemically in 3-µm FFPE sections of tumor tissue and matching bone metastases using the anti-human HER2 antibody (EP1045Y, Abcam, Cambridge, UK). For epitope retrieval, FFPE sections were incubated in a steamer in Tris/EDTA buffer (pH 9) for 20 min. The tissue slides were cooled down, rinsed with water, marked with a hydrophobic slide marker, and washed three times with TBST (Tris-buffered saline + 0.1% Tween^®^ 20). Next, the slides were blocked with Dako REAL peroxidase blocking solution (Agilent Technologies, Santa Clara, CA, USA) at room temperature for 15 min, washed three times with TBST, and blocked with 40% goat serum at room temperature for 10 min. Then, the slides were incubated with the anti-HER2 rabbit EP1045Y monoclonal IgG1 antibody (0.5 µg/mL), followed by staining using the Dako Envision^®^+ System-HRP anti-rabbit labeled polymer and the Dako DAB visualization system (both from Agilent). HER2 staining was scored by a trained scientist as intensity level (0, 1+, 2+, or 3+) relative to cell line standards. Human breast cancer cell lines with known HER2 expression statuses, including SK-BR-3 (H-score 3+), MDA-MB-453 (H-score 2+), BT-20 (H-score 1+), and MDA-MB-231 (HER2-negative, H-score 0), were stained in parallel as reference. HER2 expression was evaluated as “focal” in samples where less than 50% of the tumor cells were HER2-positive and as “homogenous” in samples with >50% of tumor cells being HER2-positive.

### 2.13. Statistical Analyses

Statistical analyses were performed using the statistical software SAS (version 9.2) or R (version 3.2.3 or newer, www.r-project.org, accessed on 20 October 2016) [47]. In the KPL-4 and Calu-3 in vivo efficacy studies, data were analyzed using one-way Analysis of Variance (ANOVA), followed by Tukey’s test. In the BT-474 in vivo efficacy study, body weight, biomarker, and radiography curves, and also curves of end-point data from different sacrifice time points were analyzed using mixed models and model contrasts. The curves were analyzed both as relative values (percentage of the baseline value) and absolute values. Whenever appropriate, the baseline value before the start of dosing was used as a covariate in the models with absolute values. In both cases, the obtained *p*-values were adjusted for multiple comparisons.

## 3. Results

### 3.1. HER2-TTC Shows Specific and Potent Binding in Various HER2-Expressing Cancer Cells

The HER2-TTC (Figure 1B) was prepared through conjugation of the 3,2-HOPO-chelator via amide bond formation to lysine side-chain amino groups on the humanized HER2 mAb and subsequent radiolabeling with thorium-227. The binding properties of the HER2 mAb and HER2-ACC were determined via flow cytometry in various cancer cell lines with different levels of HER2 expression, including BT-474 and KPL-4 breast cancer and Calu-3 lung cancer cells (Table 1, Figure 1C–E). HER2 mAb and HER2-ACC binding was shown to be specific for HER2 with nanomolar EC_50_ values (Table 1). The radiolabeled HER2-TTC was shown to retain its binding potency upon storage at room temperature for 48 h with a median IRF value of 77% (Figure 1F). Complete blocking (0% binding) of HER2-TTC was achieved when an excess of cold (unlabeled) naked HER2 antibody (0.6 mg/mL) was used to saturate all available HER2 antigens in BT-474 cells. Taken together, these results indicated that conjugation or radiolabeling did not affect the binding properties of the HER2 mAb.

### 3.2. HER2-TTC Reduces Cell Viability and Induces DNA Double Strand Break Formation and Cell Cycle Arrest In Vitro

**Figure 2:**
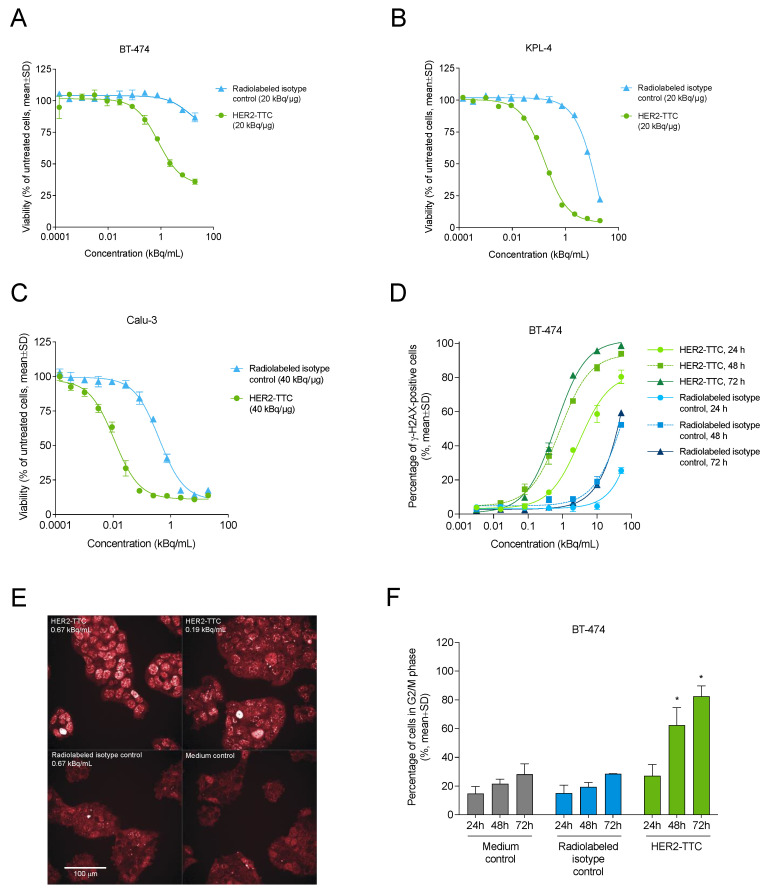
In vitro characterization and the mode of action of HER2-TTC. (**A**–**C**) Viability of (**A**) BT-474 breast cancer, (**B**) KPL-4 breast cancer, and (**C**) Calu-3 lung cancer cells after treatment with HER2-TTC or radiolabeled isotype control at 20 kBq/μg (BT-474, KPL-4) or 40 kBq/μg (Calu-3) (*n* = 3). (**D**) Induction of DNA double strand breaks (DSBs) in BT-474 cells exposed to 0.07–50 kBq/mL HER2-TTC or radiolabeled isotype control for 24, 48, or 72 h, as determined by means of detection of H2AX protein phosphorylation (γ-H2AX; *n* = 3). (**E**) Representative fluorescent microscopy images of γ-H2AX staining in BT-474 cells treated with medium, HER2-TTC, or radiolabeled isotype control for 72 h. The scale bar indicates 100 μm and is representative for all images. (**F**) Analysis of cell cycle arrest in BT-474 cells after treatment with 40 kBq/μg HER2-TTC or radiolabeled isotype control for 24, 48, or 72 h (*n* = 2–4). Statistical analysis was performed using an unpaired *t*-test. * *p* < 0.05 vs. vehicle.

The in vitro cytotoxicity and mode of action of HER2-TTC were investigated in various cancer cell lines. Exposure to HER2-TTC labeled with thorium-227 at 20 or 40 kBq/μg resulted in a dose-dependent reduction of BT-474 (Figure 2A), KPL-4 (Figure 2B), and Calu-3 (Figure 2C) cell viability with IC_50_ values of 0.02–1.8 kBq/mL (Table 1). In BT-474 cells, HER2-TTC induced the formation of DNA double strand breaks (DSBs) in a time- and dose-dependent manner (Figure 2D,E) and also caused G2/M cell cycle arrest (Figure 2F). In contrast, the radiolabeled isotype control showed only minor effects at the largest dose and/or longest exposure time.

### 3.3. HER2-TTC Shows Dose-Dependent Antitumor Efficacy in Subcutaneous KPL-4 and Calu-3 Xenograft Models

**Figure 3:**
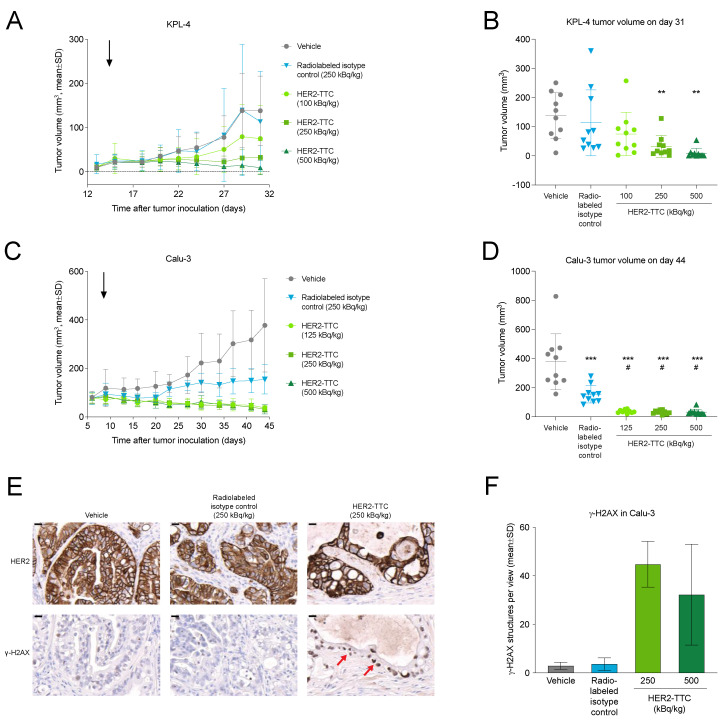
In vivo efficacy of HER2-TTC in the subcutaneous KPL-4 breast and Calu-3 lung cancer xenograft models. (**A**) Growth curves of KPL-4 tumors in female athymic nude mice (*n* = 10) treated with a single i.v. injection of vehicle, radiolabeled isotype control (250 kBq/kg), or HER2-TTC (100, 250, or 500 kBq/kg, at a total antibody dose of 0.14 mg/kg) on day 14 after tumor inoculation (treatment day indicated with a black arrow). (**B**) Tumor volumes of individual KPL-4 tumors shown in (**A**) on day 31. (**C**) Growth curves of Calu-3 tumors in female NMRI nude mice (*n* = 10) treated with a single i.v. injection of vehicle, radiolabeled isotype control (250 kBq/kg), or HER2-TTC (125, 250, or 500 kBq/kg, at a total antibody dose of 0.14 mg/kg) on day 8 after tumor inoculation (treatment day indicated with a black arrow). (**D**) Tumor volumes of individual Calu-3 tumors shown in (**C**) on day 44. (**E**) HER2 expression and γ-H2AX levels in Calu-3 tumors treated with vehicle, radiolabeled isotype control (250 kBq/kg), or HER2-TTC (250 kBq/kg) (*n* = 3). IHC analysis was performed at the end of the study, on day 37 after treatment. γ-H2AX staining indicative of DNA damage is indicated with red arrows. Magnification 20x; scale bars indicate 20 μm. (**F**) Quantification of the γ-H2AX staining shown in (**E**) (*n* = 3). Statistical analyses were performed using one-way ANOVA followed by Tukey’s test. ** *p* < 0.01; *** *p* < 0.001 vs. vehicle. ^#^ *p* < 0.05 vs. radiolabeled isotype control.

First, the in vivo efficacy of HER2-TTC was first assessed in the s.c. KPL-4 breast cancer and Calu-3 lung cancer xenograft models. In the KPL-4 model, HER2-TTC at 100, 250, or 500 kBq/kg showed dose-dependent antitumor efficacy with treatment/control ratios (T/Cs) of 0.54, 0.23, and 0.07, respectively (Figure 3A,B). In the Calu-3 model, treatment with HER2-TTC at all doses (125, 250, or 500 kBq/kg) resulted in almost complete tumor eradication with T/Cs of 0.10, 0.08, and 0.08, respectively (Figure 3C,D). No body weight loss was observed in any of the treatment groups, indicating that the treatments were well tolerated.

To investigate the in vivo mode of action of HER2-TTC, HER2 expression and H2AX phosphorylation (γ-H2AX) were determined in Calu-3 tumors at the end of the study on day 37 after treatment with 250 kBq/kg HER2-TTC or radiolabeled isotype control. Decreased HER2 staining (Figure 3E) and elevated γ-H2AX levels indicative of DNA damage (Figure 3E,F) were detected in HER2-TTC-treated Calu-3 tumors but not in vehicle or radiolabeled isotype control-treated tumors.

### 3.4. HER2-TTC Inhibits Intratibial Tumor Growth and Tumor-Induced Abnormal Bone Formation in a BT-474 Mouse Model Mimicking Breast Cancer Metastasized to Bone

**Figure 4:**
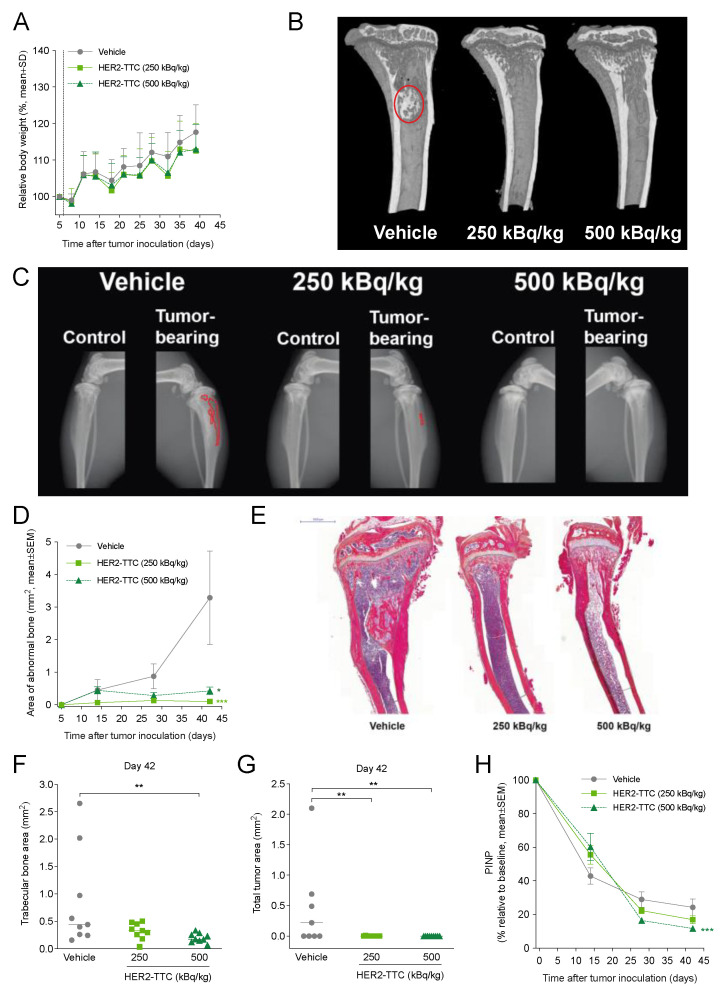
In vivo efficacy of HER2-TTC in the intratibial BT-474 mouse model mimicking breast cancer-induced metastatic bone disease. Mice were treated with a single i.v. injection of vehicle or HER2-TTC (250 or 500 kBq/kg, at a total antibody dose of 0.14 mg/kg) on day 6 after tumor inoculation. (**A**) Relative body weights of BT-474 tumor-bearing mice treated with vehicle or HER2-TTC (*n* = 9–12/group). Values are shown as percentages of initial body weights on day 5 after tumor inoculation. The dashed line indicates the day of treatment as a single injection. (**B**) Representative micro-CT images of tumor-bearing tibias of each treatment group at sacrifice on study day 42. One representative tibia is shown from each treatment group. Tumor-induced abnormal bone changes in the vehicle group are indicated with a red circle. No such foci were observed in HER2-TTC-treated mice. (**C**) Representative X-ray images of control or BT-474 tumor-bearing tibias of each treatment group at sacrifice on study day 42. Tibias of one animal per each group are shown. Red line delineates the area of tumor-induced changes in bone. (**D**) Tumor-induced abnormal bone changes determined in the tumor-bearing tibia of vehicle or HER2-TTC-treated mice using X-ray imaging and image analysis software. * *p* < 0.05; *** *p* < 0.001 vs. vehicle. (**E**) Representative histological images from hematoxylin and eosin (H&E)- and Orange G-stained sections of BT-474-inoculated tibias at sacrifice on study day 42. One tibia is shown per each treatment group. Scale bar represents 1000 μm. B, bone; T, tumor; BM, bone marrow. (**F**) Trabecular bone area including areas of tumor-induced bone growth measured in histological sections on study day 42 (*n* = 9/group). Horizontal lines indicate median values. ** *p* < 0.01 vs. vehicle. (**G**) Total tumor area measured in histological sections of BT-474-inoculated tibias on study day 42 (*n* = 9/group). Horizontal lines indicate median values. ** *p* < 0.01 vs. vehicle. (**H**) Serum bone formation marker PINP relative to baseline values in BT-474 tumor-bearing mice (*n* = 9/group). *** *p* < 0.001 vs. vehicle. All statistical analyses were performed using mixed models and model contrasts.

Next, the in vivo efficacy of HER2-TTC was assessed in the intratibial BT-474 mouse model mimicking breast cancer metastasized to bone. HER2-TTC was administered at a single i.v. dose of 250 or 500 kBq/kg. The treatments were well tolerated as the mean body weights of the treatment groups did not differ from the vehicle-treated group (Figure 4A). BT-474 tumor-induced abnormal bone formation was clearly observed in the tibias of vehicle-treated mice, as shown in micro-CT (Figure 4B) and X-ray (Figure 4C) imaging at sacrifice on day 42. Abnormal bone growth was not observed in any of the HER2-TTC-treated mice. The area of tumor-induced abnormal bone formation increased steadily over time in the vehicle group, whereas both doses of HER2-TTC inhibited the growth markedly (Figure 4D). In line with these findings, histological evaluation of tumor-bearing tibias at sacrifice (Figure 4E) demonstrated a smaller trabecular bone area (Figure 4F) and total tumor area (Figure 4G) in the HER2-TTC-treated mice compared to the vehicle-treated control group. The absence of tumor-induced abnormal bone formation in HER2-TTC-treated mice at 500 kBq/kg was also reflected in lower serum levels of the bone formation marker PINP as compared to vehicle control (Figure 4H), and this effect could be observed already on day 28. Taken together, treatment with a single i.v. dose of HER2-TTC at 250 or 500 kBq/kg resulted in marked inhibition of intratibial tumor growth and associated tumor-induced abnormal bone formation in the HER2-positive BT-474 mouse model mimicking breast cancer with bone metastases. In all experiments, both doses were equally effective since no statistical differences were observed between the two treatment groups.

### 3.5. HER2-TTC Accumulates in Bone at Sites of Active Bone Turnover

**Figure 5:**
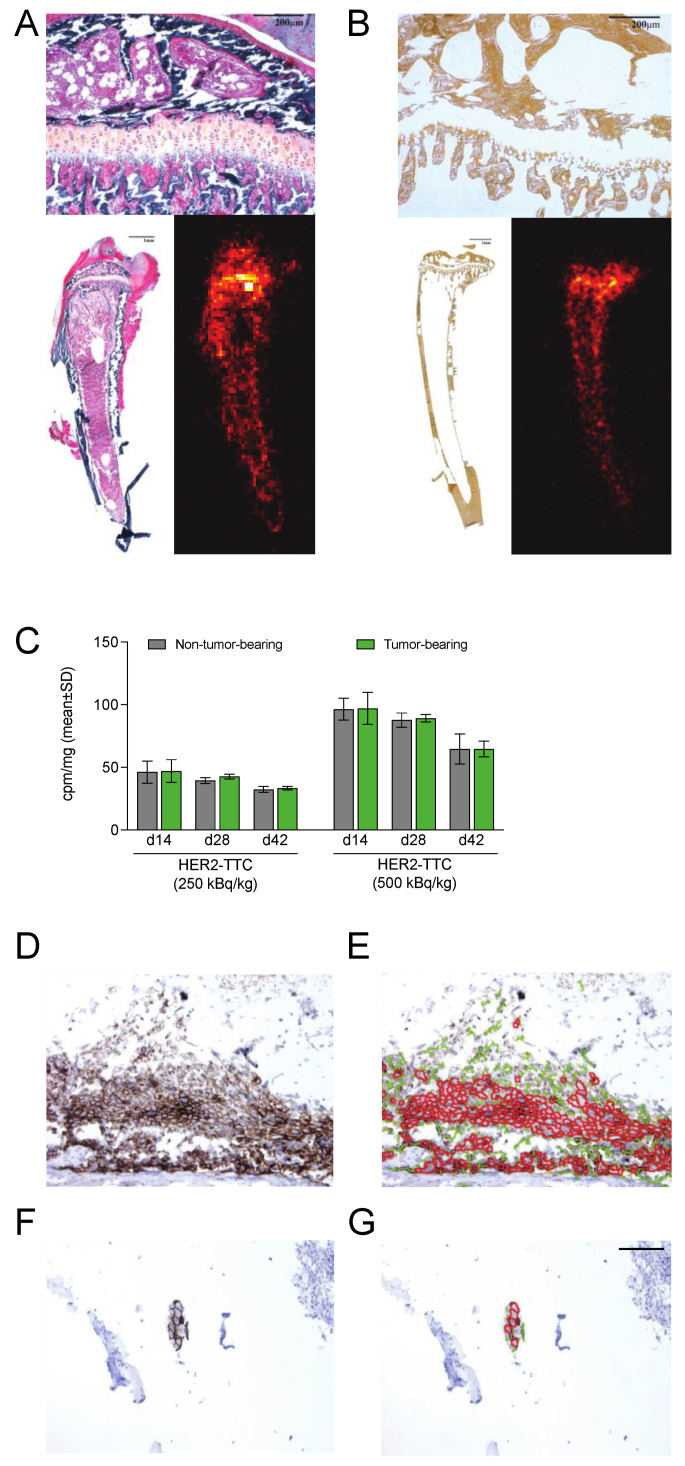
HER2-TTC accumulation to bone and HER2 expression in the intratibial BT-474 mouse model. (**A**) Trichrome Masson-Goldner staining of BT-474 tumor-bearing tibias collected 14 days after treatment with 500 kBq/kg HER2-TTC at 20× (upper panel, scale bar indicates 200 μm) and 2.5× (lower left panel, scale bar represents 1 mm) magnification and the corresponding alpha camera image (lower right panel). (**B**) Von Kossa staining of BT-474 tumor-bearing tibias collected 14 days after treatment with 500 kBq/kg HER2-TTC at 20× (upper panel, scale bar represents 200 μm) and 2.5× (lower left panel, scale bar indicates 1 mm) magnification and the corresponding alpha camera image (lower right panel). (**C**) Incorporated radioactivity relative to tibia weight (cpm/mg) in non-tumor-bearing and BT-474 tumor-bearing tibias upon treatment with 250 or 500 kBq/kg HER2-TTC. Tibias were collected 14, 28, or 42 days (d) after tumor inoculation and measured via gamma counting. (**D**) A representative image of HER2 expression in a bone tissue sample from a vehicle-treated mouse on day 42 after tumor inoculation (score 3+). (**E**) HER2 staining analysis of the sample described in panel **D** using the ImmunoMembrane web application. Red color indicates complete and strong HER2 staining, whereas green color indicates incomplete or weak HER2 staining. (**F**) HER2 expression scored as 2+/3+ in a bone tissue sample from a HER2-TTC-treated (500 kBq/kg) mouse on day 28 after tumor inoculation (the only sample in which tumor growth was detected on day 28 [*n* = 3] or 42 [*n* = 9] after tumor inoculation). (**G**) HER2 staining analysis of the sample described in panel **F** using the ImmunoMembrane web application. The scale bar in panel **G** indicates 100 μm and is representative for panels **D**–**F**. HER2 expression in BT-474 tumors was visualized using the SP3 rabbit monoclonal antibody. The HER2 status of the tumors was evaluated based on the membrane staining completeness and intensity according to the ASCO CAP 2018 guidelines [46] and analyzed using the ImmunoMembrane web application (https://biii.eu/immunomembrane, accessed on 4 August 2016). Classification/score for HER2 expression: 0/1+ = negative, 2+ = equivocal, 3+ = positive.

To evaluate the accumulation of the alpha-emitting HER2-TTC and its decay product radium-223 in bone, non-tumor-bearing and BT-474 tumor-bearing tibias were collected 14 days after treatment with HER2-TTC at 250 kBq/kg or 500 kBq/kg and analyzed via alpha camera imaging. Bone calcification was assessed in von Kossa-stained tissue sections. As expected, accumulation of HER2-TTC and radium-223, as detected via alpha camera imaging, was observed at the sites of active bone turnover, such as the epiphysis, where the growth plate is located and where the calcification of the newly formed bone is in progress (Figure 5A,B). No difference between the tumor-bearing and non-tumor-bearing tibias was observed.

Gamma counting of the tibias of HER2-TTC-treated mice showed a progressive increase in the incorporated radioactivity (cpm/mg of tibia) in both non-tumor-bearing and BT-474 tumor-bearing tibias 28 and 42 days after treatment with 250 or 500 kBq/kg HER2-TTC. In addition, increased radioactivity was detected in tumor-bearing tibias compared with non-tumor-bearing tibias on days 28 and 42 after treatment with 250 kBq/kg HER2-TTC (Figure 5C).

### 3.6. HER2 Is Expressed in Samples from the BT-474 Mouse Model Mimicking Bone Metastases

To confirm HER2 expression in BT-474 tumors, all inoculated tibias from BT-474 tumor-bearing mice were collected for IHC analysis. In five out of nine BT-474 tumor samples from vehicle-treated mice, HER2-positive tumor growth was observed and given a score of 3+ (Figure 5D,E). In BT-474 tumor samples from mice treated with 500 kBq/kg HER2-TTC, HER2 expression was also positive (score 2+/3+), but only small tumor foci were present in one sample on day 28 (*n* = 3) (Figure 5F,G). On day 42, no tumor foci were present in any of the samples (*n* = 9).

### 3.7. HER2 Expression Is Detected in Bone Metastasis Samples from HER2-Positive Breast Cancer Patients

**Figure 6:**
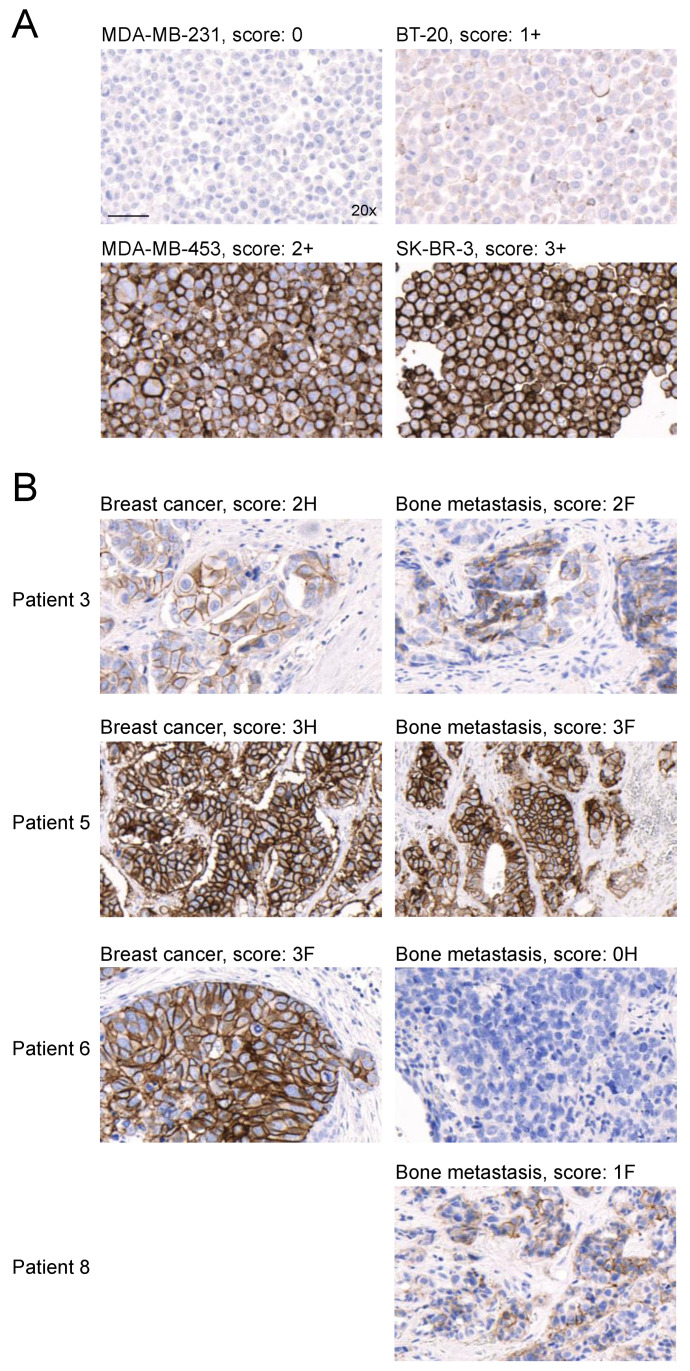
HER2 expression in matched primary breast cancer and bone metastasis samples from HER2-positive breast cancer patients. (**A**) HER2 expression in MDA-MB-231 (score 0), BT-20 (score 1+), MDA-MB-453 (score 2+), and SK-BR-3 (score 3+) breast cancer cells. HER2-positive breast cancer cells were stained as reference. (**B**) Representative images of HER2 IHC analyses in breast cancer tissue and matching bone metastasis samples from four patients with HER2-positive primary breast cancer. Brown color indicates HER2 expression. A magnification level of 20× was used. The scale bar indicates 50 μm and applies to all images.

Finally, HER2 expression was determined via IHC in seven HER2-positive breast cancer patients. Primary tumor samples and matched bone metastases from the same patients were analyzed and samples from HER2-positive breast cancer cell lines with different levels of HER2 expression were stained as reference (Figure 6A). In six out of seven patients (86%), both primary tumor and bone metastasis samples showed HER2 staining intensity ≥1, and the intensity score difference between the primary tumor and the respective bone metastasis samples was not more than 1 (Table 2, Figure 6). Clearly different levels of HER2 expression between the primary tumor and bone metastasis samples were observed only for patient 6. However, only a small cluster of HER2-positive cells was observed in the primary tumor sample of this patient and the majority of the tumor cells were HER2-negative, matching the result of the metastatic sample (Figure 6B). One additional bone metastasis sample from a HER2-positive breast cancer patient who previously underwent trastuzumab-based therapy was investigated (patient 8). This patient showed focal HER2 staining with an intensity score of 1 (Figure 6B).

## 4. Discussion

HER2 is one of the most validated targets for the treatment of breast and gastric cancer and it is used both as a prognostic and as a predictive biomarker [30]. Currently, the first-line treatment of HER2-positive patients relies heavily on the use of trastuzumab and pertuzumab in combination with systemic chemotherapy [33]. Although patients initially respond to HER2-targeting treatments, most patients eventually develop resistance via primary or acquired mechanisms [35,48], and thus more effective therapeutic approaches are needed. Indeed, several new antibody–drug conjugates (ADCs) and monoclonal antibodies for the treatment of HER2-positive metastatic breast cancer have recently been approved or are currently being tested in phase II/III trials, including the anti-HER2 ADC Enhertu (DS-8201) [49], the anti-HER2 Fc-optimized monoclonal antibody margetuximab [50], and the HER2-targeted tyrosine kinase inhibitors tucatinib and neratinib [32].

Over 70% of patients with metastatic breast cancer develop bone metastases [51,52], and they are more commonly observed in cancers with HER2 overexpression than in HER2-negative cancers [53]. HER2 overexpression has been identified in 15–30% of breast cancer patients [54,55]. There is still some ambiguity in how well the HER2 status of the primary breast cancer reflects that of its corresponding metastasis [56]. For example, Aurilio et al. demonstrated that in 92% of breast cancer patients, the HER2 status was similar in the primary breast tumor and its paired bone metastases [57], whereas Rack et al. reported that in approximately 69% of patients, the HER2 status of the primary tumors correlated with the HER2 status of the disseminated tumor cells collected from the bone marrow [58]. These observations are in line with our results, where in 86% of patients, both primary tumor bone metastasis samples showed HER2 staining intensity ≥1+, and the intensity score difference between the paired samples was not more than 1. Nevertheless, in terms of precision medicine, HER2-targeted treatments should be given to patients after confirming HER2 expression in bone metastases, not relying on the initial diagnosis, which is based on HER2 expression evaluation of the primary tumor. It has been shown that more than 10% of patients with HER2-negative primary breast cancer may still benefit from HER2-targeted treatment [59], indicating that HER2-negative patients may have metastases that are in fact HER2-positive [60] or that the primary diagnosis is false due to limitations in the IHC staining or in successfully obtaining a representative biopsy.

In contrast to beta emitters, alpha-emitting radionuclides like thorium-227 and its decay product (Figure 1A) radium-223 have a higher LET and a shorter path length (50–80 μm in tissue), and are therefore more efficacious and induce less off-target toxicity to the adjacent normal tissues [23]. Radium-223 (Xofigo^®^) is the first-in-class alpha-emitting radiopharmaceutical authorized for the treatment of metastasized CRPC [13]. Radium-223 homes to sites of active bone turnover, such as bone metastases [15], and it has demonstrated biological activity in breast cancer patients with bone metastases in early clinical trials [61,62]. Due to the lack of efficient chelator systems for radium-223, manufacturing antibody conjugate complexes with thorium-227 has become an attractive alternative [10,18].

Here, we describe the preclinical characterization of the in vitro and in vivo properties of a novel TAT, HER2-TTC, as well as its antitumor efficacy in various HER2-positive human xenograft models, including the s.c. KPL-4 breast cancer and Calu-3 lung cancer mouse models and the intratibial BT-474 mouse model mimicking bone-metastasized breast cancer. In vitro, the binding potency of HER2-TTC was comparable to the HER2-targeting antibody, indicating that conjugation and radiolabeling with the thorium-227-complexing 3,2-HOPO chelator did not impair its binding properties to the target antigen. Accordingly, the previously reported [29] in vitro stability of HER2-TTC was also confirmed here. Furthermore, we observed a specific and dose-dependent reduction in cell viability when KPL-4, Calu-3, or BT-474 cells were exposed to HER2-TTC. Thorium-227 has a high LET and only 3–5 alpha particle hits per cell are needed to induce DNA DSBs leading to cell death [2,4]. Indeed, our mode-of-action studies showed that HER2-TTC treatment increased the amount of DSBs and induced G2/M cell cycle arrest in BT-474 cells in vitro. Moreover, elevated γ-H2AX levels indicative of DNA damage were also detected in HER2-TTC-treated Calu-3 tumors ex vivo. Similar results have been demonstrated for radium-223 [3,45] and various TTCs in different preclinical cancer models [10].

We have previously demonstrated preclinical efficacy for several TTCs binding to a variety of tumor antigens, including PSMA-TTC in prostate cancer [24,25]; CD33-TTC in acute myeloid leukemia [2]; CD70-TTC in kidney cancer [23]; FGFR2-TTC in colorectal, gastric and triple-negative breast cancer [11]; MSLN-TTC in breast, colorectal, lung, ovarian and pancreatic cancer [22,27,28]; and HER2-TTC in colorectal, breast, ovarian, gastric and lung cancer [26,29]. Here, HER2-TTC showed dose-dependent in vivo antitumor efficacy in the s.c. KPL-4 breast cancer and Calu-3 lung cancer models. In the intratibial BT-474 model mimicking bone-metastasized breast cancer, HER2-TTC at both 250 and 500 kBq/kg effectively prevented both tumor growth and tumor-induced abnormal bone growth as analyzed via histology and micro-CT or X-ray imaging, respectively. The marked efficacy of HER2-TTC to reduce tumor-induced abnormal bone growth was also demonstrated through a lower trabecular bone area in HER2-TTC-treated mice compared with vehicle-treated mice. Decreasing levels of serum PINP, a systemic bone formation marker, were in line with these findings. Overlapping accumulation of both HER2-positive cells and alpha radiation were noted in the areas of abnormal bone changes. Gamma counting of the tibias of HER2-TTC-treated mice showed a progressive increase in the incorporated radioactivity in both non-tumor-bearing and BT-474 tumor-bearing tibias 28 and 42 days after tumor inoculation, possibly due to the decay of thorium-227 to radium-223, followed by radium-223 accumulation in bone. In addition, increased radioactivity was detected in tumor-bearing tibias compared with non-tumor-bearing tibias on days 28 and 42. This could be explained by the uptake of HER2-TTC into residual tumors, and/or the uptake of radium-223 into bone, especially in areas of abnormal bone growth [45].

The marked antitumor efficacy observed in the BT-474 bone metastasis model could be explained by two separate features of HER2-TTC that allow double-targeting of HER2-positive breast cancer bone metastases. Firstly, HER2-TTC directly binds to the HER2 receptor, thereby specifically targeting its alpha radiation to HER2-positive cancer cells. Secondly, thorium-227 (half-life 18.7 days) decays to radium-223 (half-life 11.4 days), which is a calcium-mimetic previously shown to reduce tumor-induced abnormal bone formation [15,16,17]. Therefore, HER2-TTC possesses a dual mode of action through inhibiting both tumor growth and tumor-induced abnormal bone growth as demonstrated in the BT-474 breast cancer model mimicking the bone-metastatic disease.

HER2-TTC was well tolerated in all in vivo models tested, because no differences in mean body weights were found between the treatment and control groups and no macroscopic findings were observed at autopsy.

## 5. Conclusions

HER2-TTC, a thorium-227-labeled HER2-targeting alpha therapeutic, induced cytotoxicity and cell cycle arrest in HER2-positive cancer cells in vitro and induced the formation of DSBs both in vitro and in vivo. HER2-TTC also demonstrated dose-dependent in vivo antitumor efficacy in subcutaneous xenograft tumor models. In a mouse model mimicking breast cancer metastasized to bone, HER2-TTC inhibited tumor growth and tumor-induced abnormal bone growth in vivo via its dual mode of action of HER2 binding and the bone-seeking properties of the radioactive decay product, radium-223. In addition, HER2 expression levels were demonstrated to be similar in the majority of matched patient samples of primary breast cancer and bone metastases. In conclusion, these preclinical results support the development of HER2-TTC as a novel TAT for the treatment of patients with HER2-positive metastatic bone disease. HER2-TTC is currently being evaluated in a phase I dose escalation study (NCT04147819).

## 6. Patents

This section is not mandatory but may be added if there are patents resulting from the work reported in this manuscript.

## Figures and Tables

**Table 1:** In vitro binding of HER2 mAb and HER2-ACC and cytotoxicity of HER2-TTC in various cancer cell lines.

Cell Line	HER2 Expression Level (ABC)	In Vitro Binding EC_50_ (nM, Mean ± SD)		In Vitro Cytotoxicity	
		HER2 mAb	HER2- ACC	Specific Activity (kBq/µg)	IC_50, HER2-TTC_ (kBq/mL, Mean ± SD)
BT-474	550,000	2.7	2.1	40	1.8 ± 1.2
				20	0.8
SK-BR-3	500,000	2.4	2.4	40	0.2 ± 0.1
				10	0.1
Calu-3	420,000	3.9 ± 0.6	5.0 ± 0.4	40	0.03 ± 0.02
				10	0.02
KPL-4	280,000	2.0 ± 0.7	2.5 ± 0.4	40	0.2 ± 0.1
				20	0.2 ± 0.1
				10	0.3 ± 0.1

ABC, antibodies bound per cell; EC_50_, half-maximal effective concentration; IC_50_, half-maximal inhibitory concentration. Experiments were performed in triplicate and repeated 1–5 times.

**Table 2:** HER2 expression in matched tumor-bone metastasis patient sample pairs.

Patient Number	Sample Type	HER2 Score (1–3)	Focal (F)/Homogeneous (H) Expression
1	Breast cancer	2	F
	Bone metastasis	3	F
2	Breast cancer	1	F
	Bone metastasis	1	H
3	Breast cancer	2	H
	Bone metastasis	2	F
4	Breast cancer	1	H
	Bone metastasis	1	F
5	Breast cancer	3	H
	Bone metastasis	3	F
6	Breast cancer	3	F
	Bone metastasis	0	H
7	Breast cancer	3	H
	Bone metastasis	2	F
8	Breast cancer	n.a.	n.a.
	Bone metastasis	1	F

n.a., not available.

## Data Availability

The data underlying this article are available in the article.
